# Real-time prediction of ROP based on GRU-Informer

**DOI:** 10.1038/s41598-024-52261-7

**Published:** 2024-01-25

**Authors:** Bingrui Tu, Kai Bai, Ce Zhan, Wanxing Zhang

**Affiliations:** 1 Hubei Key Laboratory of Drilling and Production Engineering for Oil and Gas, Yangtze University, Wuhan, China; 2 Xi’an Key Laboratory of Tight Oil (Shale Oil) Development (Xi’an Shiyou University), Xi’an, 710065 Shaanxi China; 3 School of Computer Science, Yangtze University, Jingzhou, 430023 China

**Keywords:** Computer science, Crude oil

## Abstract

Accurate ROP (rate of penetration) prediction contributes to better production task planning, ensuring efficient production line operation, and reducing production costs. ROP prediction is influenced by multiple factors, making accurate prediction challenging. Current research primarily relies on historical data for training and modeling, lacking methods for real-time ROP prediction. This paper introduces a GRU-Informer model for real-time ROP prediction. The model employs GRU (Gated Recurrent Unit) neural networks at the lower level to capture short-term correlations in drilling parameters and uses the Informer model at the top to address long-term dependencies among drilling parameters. Thus, the GRU-Informer can capture both short-term and long-term time dependencies, providing better ROP predictions. This paper constructs a dataset using historical data from a southwestern Chinese oil field for experimentation. RMSE (Root Mean Square Error), MAE (mean absolute error) and ${R}^{2}$ (Coefficient of Determination) are employed as evaluation metrics for the model. Experimental results demonstrate that the GRU-Informer outperforms traditional recurrent neural networks like LSTM (Long Short-Term Memory), GRU neural networks and Informer in real-time ROP prediction, indicating its practical value.

## Introduction

In drilling engineering, ROP is a common metric used to assess the energy efficiency of drilling operations. In recent years, scholars have made significant advancements in the field of ROP prediction by developing mathematical models, machine learning models, and deep learning models. Currently, an increasing number of scholars are using deep learning methods for ROP prediction.

Currently, scholars have achieved promising results in ROP prediction and optimization. However, there are still issues in the real-time ROP prediction field that require further research. Firstly, the current process of researching ROP prediction and optimization is offline, meaning that model training and optimization are based on historical data collection. Such models cannot provide guidance for on-site operations. Secondly, drilling sequences contain a wealth of information, such as geological data, which conventional models often fail to fully extract. In light of these issues, this paper proposes a composite model for real-time mechanical drilling speed prediction using the GRU-Informer neural network. By combining the model training and optimization processes and employing real-time data transmission and analysis, the predictive model can be updated in real-time during drilling operations, aiding engineers in making timely decisions. Building on these two challenges, this paper establishes a real-time ROP prediction model based on the GRU-Informer.

## Related work

### ROP prediction

The development of mechanical drilling speed prediction has gone through several stages, encompassing various methods and technologies. In the early stages of mechanical drilling speed prediction, it primarily relied on engineers' experience and intuition. These empirical models depended on the expertise of professionals and took into account fundamental drilling parameters, such as drill bit type, rock formation, well depth, and more.

Friedman^1^ categorize ROP prediction can be formulated as:

1 $$ROP = f(x)$$

In this context, x represents input parameters, typically various drilling parameters (such as drill bit type, drilling speed, torque, etc.), where the function f(⋅) serves as a regression function that maps the input space to the output space, i.e., $f:X \to Y$. Therefore, various methods can be applied to obtain a predictive model for ROP.

Bourgoyne^2^ laid the foundation for subsequent research by establishing a drilling rate equation through an analysis of the relationship between drilling speed and various drilling parameters. Maurer^3^ discovered that under "ideal cleanliness" conditions, drilling speed is directly proportional to rotational speed, directly proportional to the square of the drill bit weight, and inversely proportional to the sum of the square of the drill bit diameter and rock strength. Motahhari^3^ proposed a method for predicting the ROP of any PDC drill bit design based on PDM output. However, due to the complexity of factors involved in the drilling process, while these models can sometimes offer useful guidance, they cannot provide systematic data analysis and model optimization.

When researchers recognized these issues, their focus shifted towards machine learning. Initially, researchers explored the feasibility and superiority of machine learning models for ROP prediction, including SVM, RF, Logistic, and ANN neural networks^4–7^. For example, Ahmed^4^ employed SVM to predict over 400 field data points from shale formations, demonstrating that the SVM model provided predictions with lower errors and closer alignment to practical outcomes. Subsequently, scholars investigated the use of machine learning algorithms to optimize regression models or enhance artificial neural networks for ROP prediction^8–11^. For instance, Bahari^8^ utilized a General Regression Neural Network (GRNN) to predict ROP. For the inputs of the proposed models, Genetic Algorithms (GA) were employed to optimize the traditional model of Bourgoyne and Young. Manshad^9^ employed a combination of GA, Particle Swarm Optimization (PSO), and ANN methods for ROP prediction. The ANN, optimized using a hybrid GA-PSO algorithm, outperformed individual GA and PSO methods. Their research demonstrated that machine learning offers numerous advantages in ROP prediction, especially in mapping nonlinear and complex relationships.

Researchers have observed the sequential and temporal characteristics in the problem of predicting Rate of Penetration (ROP), treating it as a time-series prediction issue where the ROP prediction is regarded as a sequence ordered by depth in a well. The prediction of ROP at the current state is not only related to the information of various parameters at the current state but also connected to the information from past moments. Recurrent Neural Network (RNN) models, such as Long Short-Term Memory (LSTM) and Gated Recurrent Unit (GRU), which excel at handling sequence prediction problems, have been employed by researchers to establish ROP prediction models^12–14^. Ji et al.^13^ discovered that LSTM performs the best in predicting the drilling speed of large clusters of offshore drilling machinery. Ao^15^ applied LSTM to deep sequence problem prediction, achieving good results with a minimum absolute error of only 2.11 m/h. Liu^16^ combined LSTM with Feedforward Neural Network (FNN), demonstrating that the new model surpasses LSTM in predicting ROP. Ji et al. utilized particle swarm optimization to enhance LSTM, resulting in a 44.2% improvement in model performance compared to the unoptimized version. However, due to the inherent limitations in the model structure of RNNs, they are unable to further extract potentially valuable information from sequential data.

### Self-attention mechanism

The self-attention mechanism is a widely applied technique in deep learning, particularly achieving remarkable success in Natural Language Processing (NLP) tasks. Initially proposed in the field of computer vision, attention mechanisms were introduced by Volodymyr^17^ into Recurrent Neural Network (RNN) models for image classification. The new model exhibited significant improvements over convolutional neural networks in cluttered image classification and demonstrated the ability to track simple objects in dynamic visual control problems. Bahdanau^18^ first applied the attention mechanism in the NLP domain, employing a Seq2Seq+ Attention model for machine translation and achieving enhanced results.

While attention mechanisms have succeeded in enhancing the performance of sequence-to-sequence models, their computational cost increases with the length of the input sequence. To further improve efficiency, the Google Machine Translation team^19^ introduced the self-attention mechanism. This mechanism allows the model to consider information from all other elements when processing each element, without relying on fixed weights. Specifically, the Transformer model introduced the self-attention mechanism for the first time, achieving significant success in NLP tasks.

In recent years, the rise of pre-trained models such as BERT and GPT has further propelled the development of self-attention mechanisms. These models, pre-trained on large-scale corpora, learn universal language representations. When fine-tuned for specific tasks, they exhibit outstanding performance, validating the effectiveness of self-attention mechanisms. Additionally, in the field of regression prediction, the application of self-attention mechanisms has made significant progress. Hu^20^ proposed a network self-attention mechanism and an RNN-based time series prediction model. Through predictions on Construction Cost Index (CGI), M1, and M3 datasets, the model demonstrated superior predictive performance compared to other models.

Drilling operations typically generate sequential data, where the sequential features are reflected not only in engineering data such as mud properties but also in geological parameters that provide information about subsurface layers. The self-attention mechanism aids in capturing dependencies between different time points. Integrating the self-attention mechanism into ROP prediction models enhances the understanding of dynamic patterns within time series data.

## Model architecture design

As indicated in Sect. 1, many researchers have utilized RNNs to construct ROP prediction models; however, ROP prediction models based on RNNs may have certain limitations. RNNs are typically used for short-term predictions, and drilling data constitutes a long sequence where RNNs may not be well-suited for capturing parameter relationships within the long sequence of data. RNNs may yield satisfactory predictions when the well depth sequence is short, but they may produce larger prediction errors when the well depth sequence is extensive. Self-attention mechanisms address long-term dependencies between input and output by calculating the relevance of all data and exhibit strong long-term prediction capabilities. However, it is not particularly sensitive to short-term sequence features, leading to significant errors in the final results. In summary, a single model has its limitations. Therefore, this paper opts to combine GRU with the self-attention mechanism model Informer to address the shortcomings of a single model.

### GRU neural network

**Figure 1:**
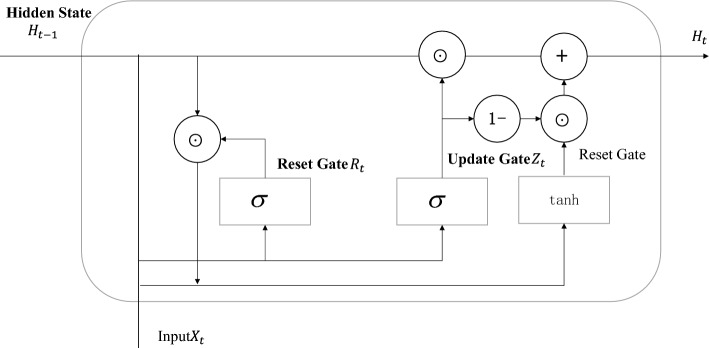
GRU structure.

Common recurrent neural networks include RNN and LSTM. LSTM possesses longer memory capabilities and generally outperforms basic RNN models in most sequence tasks. More importantly, LSTM is less prone to the vanishing gradient problem. However, the LSTM structure is relatively complex, with higher computational costs and a larger number of model parameters. GRU (Gate Recurrent Unit) is a type of recurrent neural network and is one of the simplified models of LSTM proposed by Cho et al.^21^. Therefore, the network topology of GRU is similar to that of LSTM. LSTM has three gates in its network structure: the forget gate, input gate, and output gate, whereas GRU has only two gates in its network structure: the reset gate and update gate. Structurally, GRU is a simplification compared to LSTM, making it computationally more efficient. The structure of GRU includes two key gates: the reset gate and the update gate. GRU employs these gate mechanisms to selectively update and forget information, adapting to input data at different time steps, thus enhancing its ability to capture long-term d ependencies within sequences. Figure 1 is a schematic diagram of the GRU structure:

The update gate ${z}_{t}$ determines how the new hidden state ${h}_{t}$ should inherit the previous hidden state ${h}_{t-1}$. The calculation process is formulated as:

2 $$z_{t} = \sigma (W_{z} \cdot [h_{t - 1} ,x_{t} ])$$

The reset gate ${r}_{t}$ determines whether to ignore some of the information in the previous hidden state ${h}_{t-1}$. The calculation process is formulated as:

3 $$r_{t} = \sigma (W_{r} \cdot [h_{t - 1} ,x_{t} ])$$

The new candidate hidden state $\widetilde{h}t$ is calculated based on the previous hidden state ${h}_{t-1}$ 和 and the current input ${x}_{t},$ but it has not considered the influence of the update gate. The calculation process is formulated as:

4 $$\mathop h\limits^{\sim }_{t} = \tanh (W \cdot [r_{t} \odot h_{t - 1} ,x_{t} ])$$

The final hidden state *ht* is controlled by the update gate ${z}_{t}$,which determines how much information from the new candidate hidden state $\widetilde{h}t$ and the old hidden state ${h}_{t-1}$ will be retained. Its calculation formula is formulated as:

5 $$h_{t} = (1 - z_{t} ) \odot h_{t - 1} + z_{t} \odot \mathop {h_{t} }\limits^{\sim }$$

### Informer

**Figure 2:**
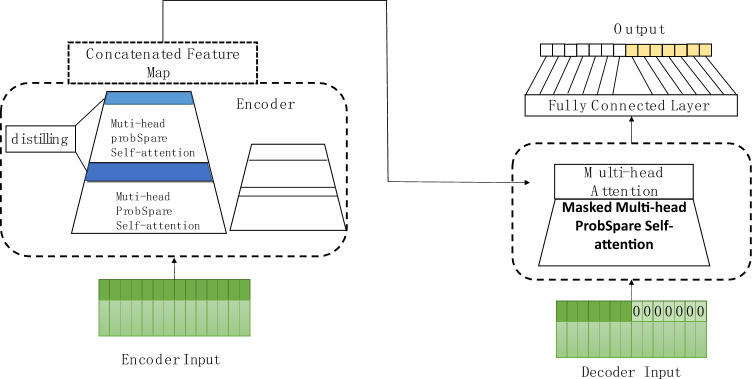
Informer structure.

Figure 2 illustrates the fundamental architecture of the Informer network, showing that Informer employs an encoder-decoder structure. In comparison to Transformer, Informer introduces the sparse self-attention mechanism as a replacement for the traditional self-attention mechanism. It also proposes the distillation self-attention mechanism to reduce the input sequence length in each layer and employs generative decoding to address the shortcomings of Transformer in time series prediction.

Both the encoder and decoder of Informer can accept data input. The encoder processes a set of long-term data sequences, while the decoder handles sequences of the same length as the predicted sequence, combined with zeros. The encoder consists of multiple sets of multihead ProbSparse self-attention layers and a distillation layer. The sparse self-attention mechanism is a variation of the self-attention mechanism, where the conventional self-attention mechanism's calculation process is formulated as:

6 $$Atten(Q,K,V) = soft\max \left( {\frac{{QK^{T} }}{\sqrt d }} \right)V$$

In this context, Query (Q) represents the query vector used to specify the focus of attention; Key (K) serves as the key vector to provide information related to the query vector, and Value (V) is the value vector that offers actual numerical information related to both the query and key. The 'd' denotes the dimension of the query, key, and value vectors. In sparse self-attention, only a few dot products contribute significantly to the main attention, while others can be disregarded. The query vector contains active and inactive queries. Dot products should be calculated for active queries, while the dot products for inactive queries are replaced by the average of the value vectors, thereby reducing computational tasks. The new self-attention is composed of active queries, and sparse self-attention is formulated as:

7 $$Atten(Q,K,V) = Soft\max \left( {\frac{{\overline{Q}K^{T} }}{\sqrt d }} \right)V$$

The purpose of the distillation operation is to shorten the input sequence length in each layer, thereby reducing memory usage. The distillation process from the j-th layer to the (j + 1)-th layer is formulated as:

8 $$X_{j + 1}^{t} = MaxPool(ELU(Conuld([X_{j}^{t} ]_{AB} )))$$

The decoder part employs the encoder mechanism, as seen in the structural diagram, it is constructed by stacking two identical multihead probability coefficient self-attention layers. This approach allows obtaining results in a single step when predicting sequences, alleviating efficiency issues in long-term predictions.

### GRU-Informer model

**Figure 3:**
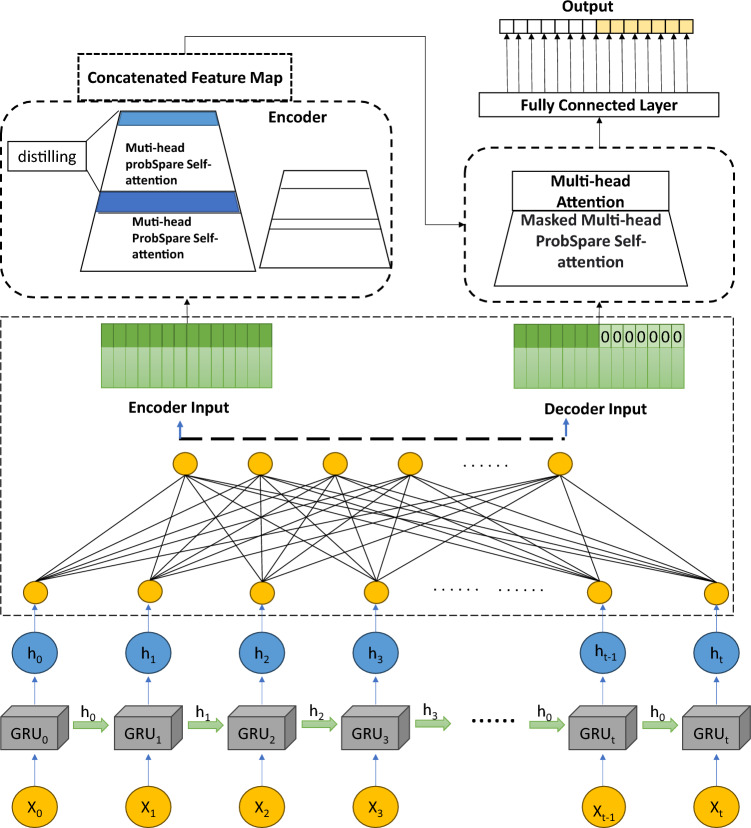
GRU-Informer structure.

Figure 3 illustrates the architecture of GRU-Informer. Drilling sequence data is fed into the GRU model using a sliding window approach. This means that the data is divided into non-overlapping windows, with each window containing a segment of continuous time series data. These windows are used to construct the input sequence for the model. The inputs of the Informer's encoder and decoder are used to receive the output of the GRU neural network. The encoder section receives a long sequence of historical data, indicating that the encoder's input is a sequence composed of multiple time windows, used to learn features of time series data. The decoder section receives data from the current time step, along with a zero sequence equal to the prediction horizon. This is done to generate predictions for the future. The zero sequence serves as a placeholder, informing the model to only consider information from the current time step during prediction. The encoder section, through multiple operations, including multi-head probabilistic sparse self-attention modules and a "distillation" mechanism module, transforms the input sequence into intermediate results. This process aids in extracting feature representations of the input data and modeling the dependencies between historical data. The decoder section initially performs multi-head probabilistic sparse self-attention operations with masks to ensure that the model focuses only on relevant parts of historical data when generating predictions. Next, the decoder utilizes the intermediate results from the encoder to perform multi-head self-attention operations, generating the prediction sequence. Finally, the data output dimensions are adjusted using a fully connected layer to obtain the prediction results.

## Methodology

This section primarily aims to introduce the data sets used in this paper and provide details of the experimental design. Additionally, this section offers information about feature engineering and data preprocessing. Section 3.1 provides a detailed account of the data sets and the data cleaning process. Section 3.2 discusses the feature selection process conducted to ensure data quality and reduce model complexity. Section 3.3 mainly covers the relevant experimental details.

### Data preparation

The data used in this study is derived from historical records of 58 wells in a southwestern oilfield in China. These data encompass 12 features, including well depth, true vertical depth, drilling speed, and hook load, among others. Three wells with similar geological parameters and comparable well types were selected for experimentation. Table 1 provides information on each well, including well names, depth ranges, and data quantities. Given the experimental goal of real-time Rate of Penetration (ROP) prediction, the data in the simulation experiment are fed into the model for prediction in real-time. Due to the real-time nature of the experiment, no filtering or other noise reduction processes were applied to the incoming real-time data. To mitigate the impact of noise on model performance, only basic data cleaning was performed on input parameters during model training. Data preprocessing for this experiment included outlier removal and missing value imputation. Outliers, typically arising from input errors or sensor malfunctions, were directly removed from the dataset. For continuous missing values, the mean of the maximum and minimum values was used for imputation. For individual missing values, the average of neighboring values was employed for imputation. This approach allowed the study to address the impact of outliers and missing values on the model's performance while simulating real-time ROP prediction under conditions resembling those encountered in the field.

**Table 1:** Partial data presentation.

Well name	Start depth (m)	End depth (m)	Data Num
Well 1	400	5112	4714
Well 2	1250	4168	2900
Well 3	401	3930	3531

### Feature selection

In oil and gas drilling, the performance of ROP prediction models is influenced by the selection and quality of input features. Different input features can provide information about various aspects of drilling operations, making their impact on ROP prediction models crucial. Different input features can provide information about various aspects of drilling operations, making their impact on ROP prediction models crucial. In this paper, the Pearson correlation coefficient is used to calculate the correlation between parameters. The Pearson correlation coefficient is a linear correlation coefficient and is also one of the most commonly used correlation measures. It is typically employed to reflect the degree of linear correlation between two variables, and its value ranges from − 1 to 1, with a larger absolute value indicating a stronger correlation.

The population relationship coefficient ρ is defined as the ratio of the covariance between two variables and the standard product of them, as formulates:

9 $$\rho_{X,Y} = \frac{{{\text{cov}} (X,Y)}}{{\sigma_{X} \sigma_{Y} }} = \frac{{E[(X - \mu_{X} )(Y - \mu_{X} )]}}{{\sigma_{X} \sigma_{Y} }}$$

**Figure 4:**
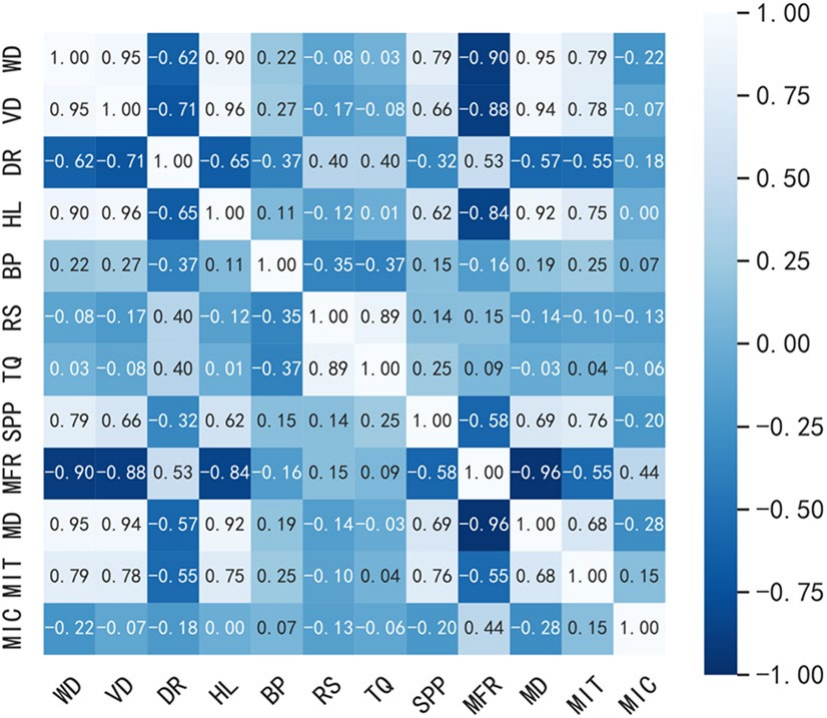
Correlation coefficient heat map.

Estimating the sample covariance and standard deviation allows for the calculation of the sample correlation coefficient, commonly denoted as $r$:

10 $$r = \frac{{\sum\nolimits_{i = 1}^{n} {(X_{i} - \overline{X})(Y_{i} - \overline{Y})} }}{{\sqrt {\sum\nolimits_{i = 1}^{n} {(X_{i} - \overline{X})^{2} } } \sqrt {\sum\nolimits_{i = 1}^{n} {(Y_{i} - \overline{Y})^{2} } } }}$$

where $\frac{{X_{i} - \overline{X}}}{{\sigma_{X} }},\overline{X}$ are the sample z-scores, $\sigma_{X}$ is the sample mean, n is the sample standard deviation, and n is the sample size. According to the Pearson method, the correlation coefficients between parameters are calculated, and their correlation heatmap is shown in Fig. 4.

Figure 4 shows that ROP is strongly correlated with well depth, vertical depth, and hook load, while the correlation of other features mostly aligns with the principles of physical drilling. However, there are also situations that contradict commonly accepted knowledge. For instance, rotational speed and drilling pressure are considered key factors influencing ROP. However, from the perspective of correlation analysis, these two features exhibit relatively weak correlations with ROP. On the other hand, despite the weak correlation between drilling fluid inlet temperature and ROP, it exhibits a relatively high correlation coefficient, which contradicts the actual drilling patterns. This is because these correlation coefficients are calculated using statistical methods, revealing mathematical relationships between data, rather than necessarily reflecting the intrinsic relationships between features. Therefore, when selecting features, a combination of feature analysis and drilling theory should be considered. In the end, this paper selects well depth, vertical depth, hook load, drilling pressure, rotational speed, torque, casing pressure, and inlet density as the input parameters for the model. The full names of the abbreviations in Fig. 4 are as indicated in Table 2.

**Table 2:** Heat map abbreviation and its full name.

Full name	Abbreviation	Full name	Abbreviation
Well depth	WD	Torque	TQ
Vertical depth	VD	Standpipe pressure	SPP
Drilling rate	DR	Mud flow rate	MFR
Bit pressure	BP	Mud density	MD
Rotary speed	RS	Mud inlet temperature	MIT
Hook load	HL	Mud inlet conductivity	MIC

### Evaluation metrics

In this paper, root mean square error (RMSE), mean absolute error (MAE) and determination coefficient (${R}^{2}$) are selected as evaluation indexes to evaluate the model.

11 $$MSE = \frac{1}{n}\sum\limits_{i = 1}^{n} {(y_{i} - \hat{y}_{i} )^{2} , \in [0, + \infty )}$$

12 $$MAE = \frac{1}{n}\sum\limits_{i = 1}^{n} | y_{i} - \hat{y}_{i} |, \in [0, + \infty )$$

13 $$R^{2} = 1 - \frac{{\sum\nolimits_{i = 1}^{n} {(y_{i} - \hat{y}_{i} )^{2} } }}{{\sum\nolimits_{i = 1}^{n} {(y_{i} - \overline{y})^{2} } }} \in [0,1]$$

In the equation, n represents the sample size, $y_{i}$ signifies the true value of the i-th sample, and $\widehat{{\text{y}}}_{i}$ represents the predicted value for the i-th sample. Mean Squared Error (MSE) assesses the model's precision at a numerical level; a smaller MSE indicates a closer match between predicted and actual values, signifying a better model fit. Mean Absolute Error (MAE) is utilized to measure the average absolute error between predicted values and true values. A smaller MAE indicates a superior model. On the other hand, R-squared (${R}^{2}$) evaluates the overall model fit from a statistical perspective, with a range between 0 and 1. The closer R-squared (${R}^{2}$) is to 1, the better the predictive model, indicating that the model's predicted values are closer to the actual measurements.

The original data undergoes data preprocessing in Sect. 3.1 and is then standardized. After standardization, the data needs to be input into the model for training. Typically, model training consists of the following steps: firstly, preprocessing data is split into a training set and a test set; then, the training set data is used to train the model, while the test set is used to assess the model's fit. Subsequently, hyperparameters are continuously adjusted, and well-fitting models are employed for ROP prediction. The above process is suitable for offline prediction but cannot be applied to real-time ROP prediction. In constructing a real-time ROP prediction model, it is not feasible to randomly split the dataset; instead, data generated during the drilling process must be continuously used for training and predicting the ROP of lower well segments.

## Analysis of experimental results

**Figure 5:**
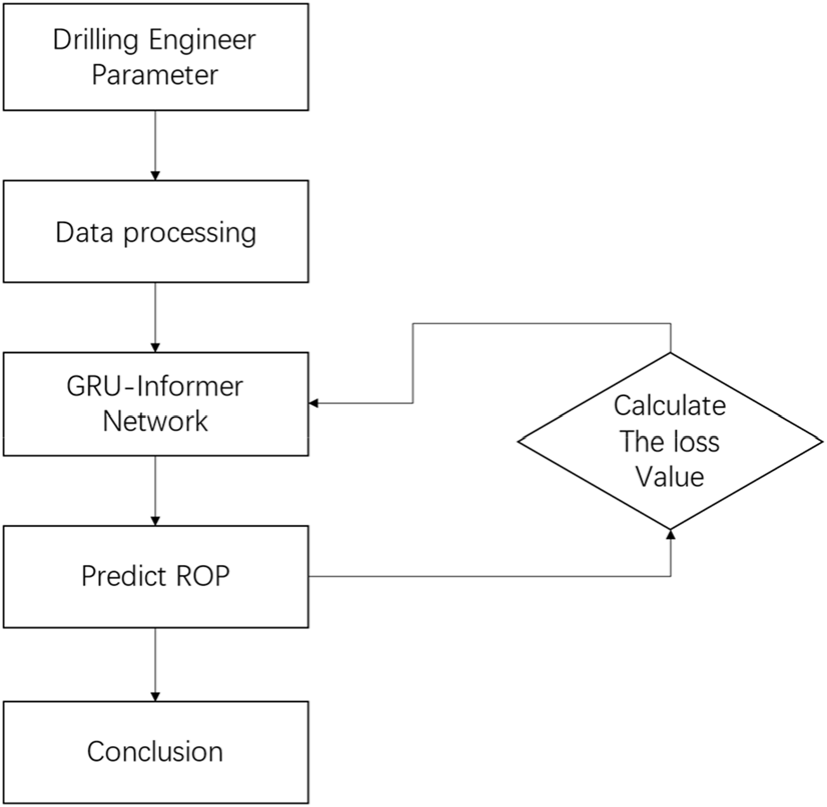
Experimental process.

This chapter primarily focuses on the design, implementation, and conclusions of the experiment, as illustrated in Fig. 5. The data processed in Chapter 3 is fed into the model for training, and the model parameters are continuously adjusted based on the variation in the loss values. The best-performing model is selected and compared with GRU, Informer, and LSTM on the test set to analyze the performance of each model. The experimental framework is built on Python 3.8 and PyTorch 1.1.1, utilizing a computer configuration with an i5 12400f.CPU and NVIDIA 2060 graphics card.

### LSTM model

**Figure 6:**
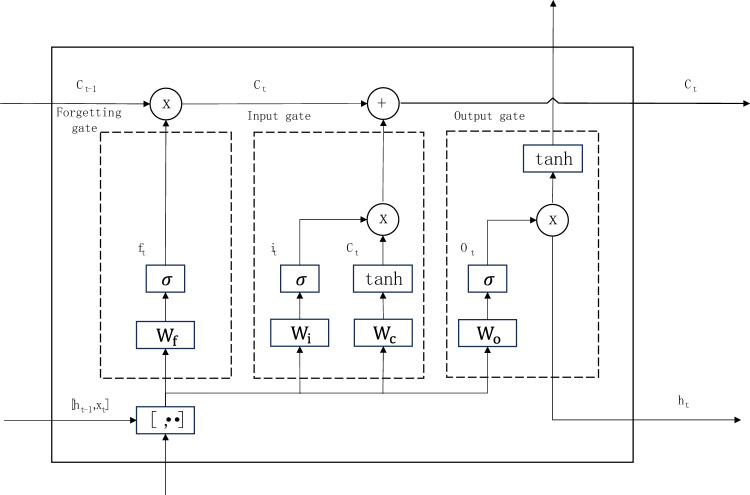
LSTM model structure.

This paper employs the traditional Long Short-Term Memory (LSTM) as a comparative model. Proposed by Hochreiter et al.^22^, LSTM has become a powerful tool for sequence modeling and finds widespread applications in natural language processing, time series analysis, and various other fields. The structure of LSTM is depicted in Fig. 6.

### Sliding input flow

The original data undergoes preprocessing in Sect. 3.1, followed by data standardization. After standardization, the data needs to be input into the model for training. Typically, the model training involves the following steps: initially dividing the preprocessed data into training and testing sets; utilizing the training set data for model training and the testing set for evaluating the model's fitting performance; continuously adjusting hyperparameters and using well-fitted models for ROP prediction. This workflow is suitable for offline prediction but is not applicable for real-time ROP prediction. In constructing a real-time ROP prediction model, it is not feasible to randomly split the dataset. Instead, the data generated during the drilling process needs to be continuously used for training and predicting the ROP of the subsequent well segments.

**Figure 7:**
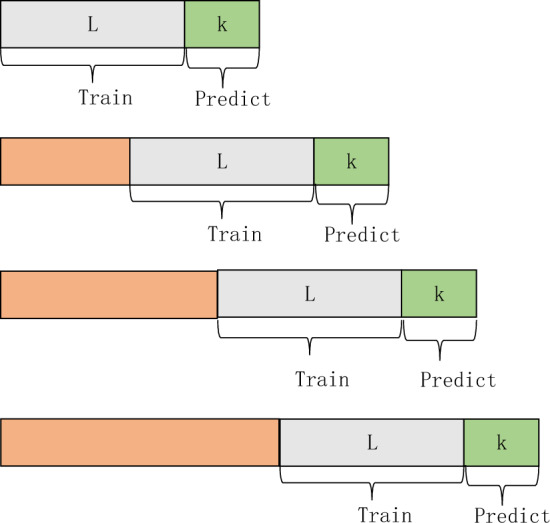
Sliding input flow.

Firstly, three wells with similar well types and geological parameters are selected from the data in Sect. 3.1 for experimentation. Among them, Well1 is used for training the model and referred to as the training well, while Well2 and Well3 are used for real-time ROP experiment verification, referred to as the testing wells. The data from the training well is input into the model for training. Subsequently, the data from the testing wells is input into the model for prediction, as illustrated in Fig. 7. The prediction model network adopts a sliding module approach, using the last L meters of data as input to predict the ROP for the next k meters. In this paper, L is set to 100, and k is set to 10. Finally, evaluation metrics are used to assess the model's prediction results.

### Selection of model superparameters

Learning rate and batch size are two crucial hyperparameters in the training of deep learning models, and they have a significant impact on the model's performance and training process. Typically, each sliding window is treated as an independent training sample, and the model parameters are updated for each window. However, a potential issue with this approach is that there may be high correlation between adjacent windows since they share many neighboring data points. In this study, multiple sliding windows are combined into a small batch to reduce the correlation between adjacent windows while still allowing the model to consider information from multiple time steps in a single update.

During the experimental process, GRU-Informer, GRU, Informer, and LSTM are set to the same input parameters, iteration count, learning rate, and batch size for a controlled comparison of their performance under a single variable condition. In practical applications, choosing appropriate learning rates and batch sizes often requires experimentation and adjustments. As shown in Table 3, different learning rates and batch sizes are employed for ROP prediction model training. The experimental results indicate that when the learning rate is 0.0001 and the batch size is 32, the model achieves the lowest Mean Absolute Error (MAE). Therefore, the subsequent experimental processes adopt the hyperparameters as shown in Table 4.

**Table 3:** Model MAE under different learning rate and different batch.

Learning rate	Batch size			
	32	64	128	256
0.0001	**10.5833**	11.6762	12.4732	13.2420
0.0005	11.3486	12.4267	12.6606	12.7562
0.005	11.8359	12.5356	12.6711	12.8259
0.001	12.1345	12.3496	12.7357	12.8573

Significant values are in bold.

**Table 4:** Hyperparameters of the model.

Epoch	Batch size	Leaning rate	NetWork layer
500	32	0.0001	128

### Result analysis

Table 5 displays the training times required for each model. From the data in the Table 5, it is evident that GRU-Informer takes more time than Informer and GRU networks but less time than the LSTM model. This is attributed to the higher computational cost of composite models compared to single models due to their increased complexity. Future efforts will be directed towards enhancing model accuracy while simultaneously reducing model complexity.

**Table 5:** Model training time.

Model	Traning time (s)
GRU-Informer	191.6
LSTM	213.8
GRU	189.6
Informer	**172.4**

Significant values are in bold.

**Figure 8:**
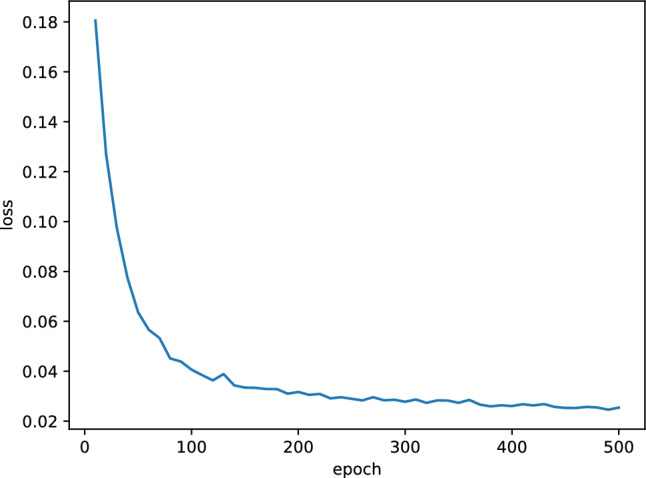
Training loss.

Figure 8 illustrates the loss change curve during the training process of the GRU-Informer model. The loss values rapidly decrease from 0 to 100 rounds, and the rate of descent slows down from 100 to 500 rounds, indicating that the model is converging. The model from the round with the lowest loss value is selected and saved for further experimental analysis.

**Figure 9:**
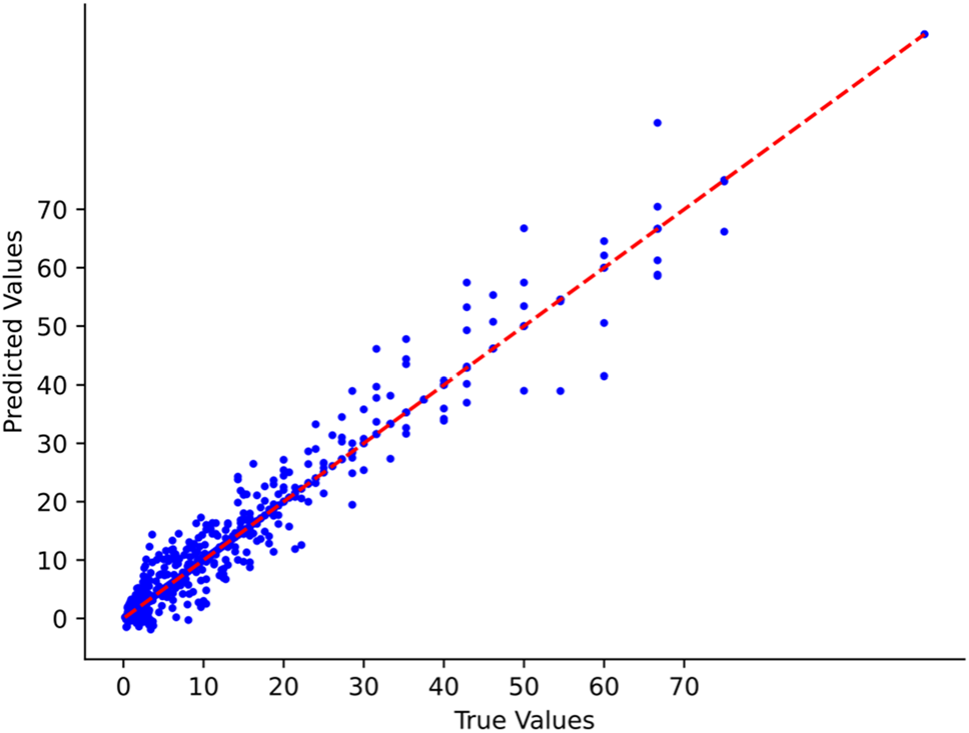
GRU-Informer predicted values and true values in Well 3.

To validate whether the GRU-Informer model exhibits superior accuracy in predicting the Rate of Penetration (ROP) compared to other models, the trained model underwent testing on the validation set and was compared with GRU, Informer, and LSTM models. Figure 9 illustrates the predictive results of the GRU-Informer model on Well 3, showing the predicted values against the actual ROP values.The model demonstrates a high level of accuracy in predicting ROP within the range of 0–40 m/h. However, a decrease in prediction accuracy is observed when ROP exceeds 40 m/h. This decline could be attributed to the rarity of instances where the drilling speed exceeds 40 m/h in the actual drilling process of this set of wells. Consequently, the limited amount of data available for training may have impacted the model's performance in the higher ROP range.

**Figure 10:**
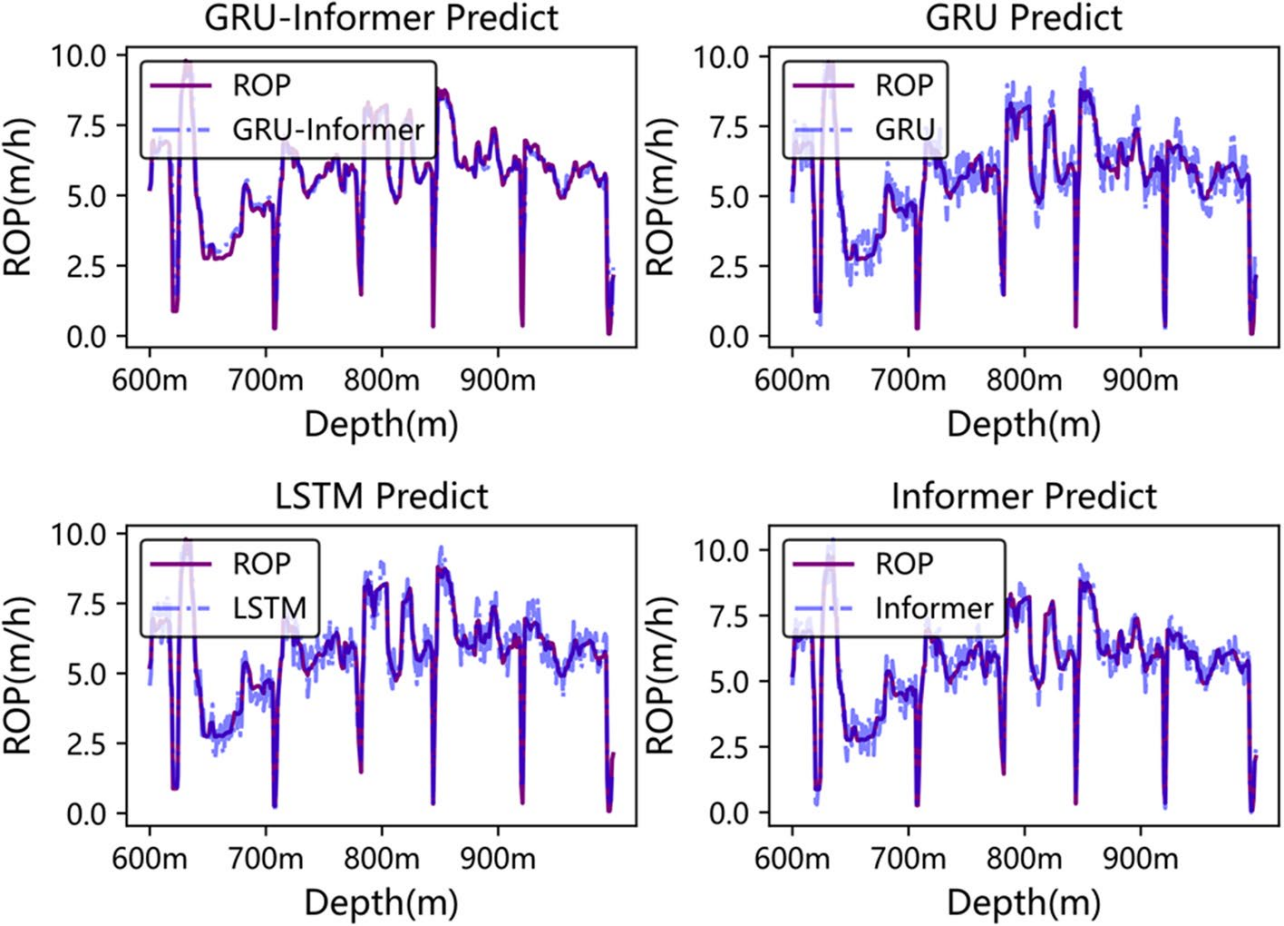
Model prediction curve of 600–1000 m in well3.

**Figure 11:**
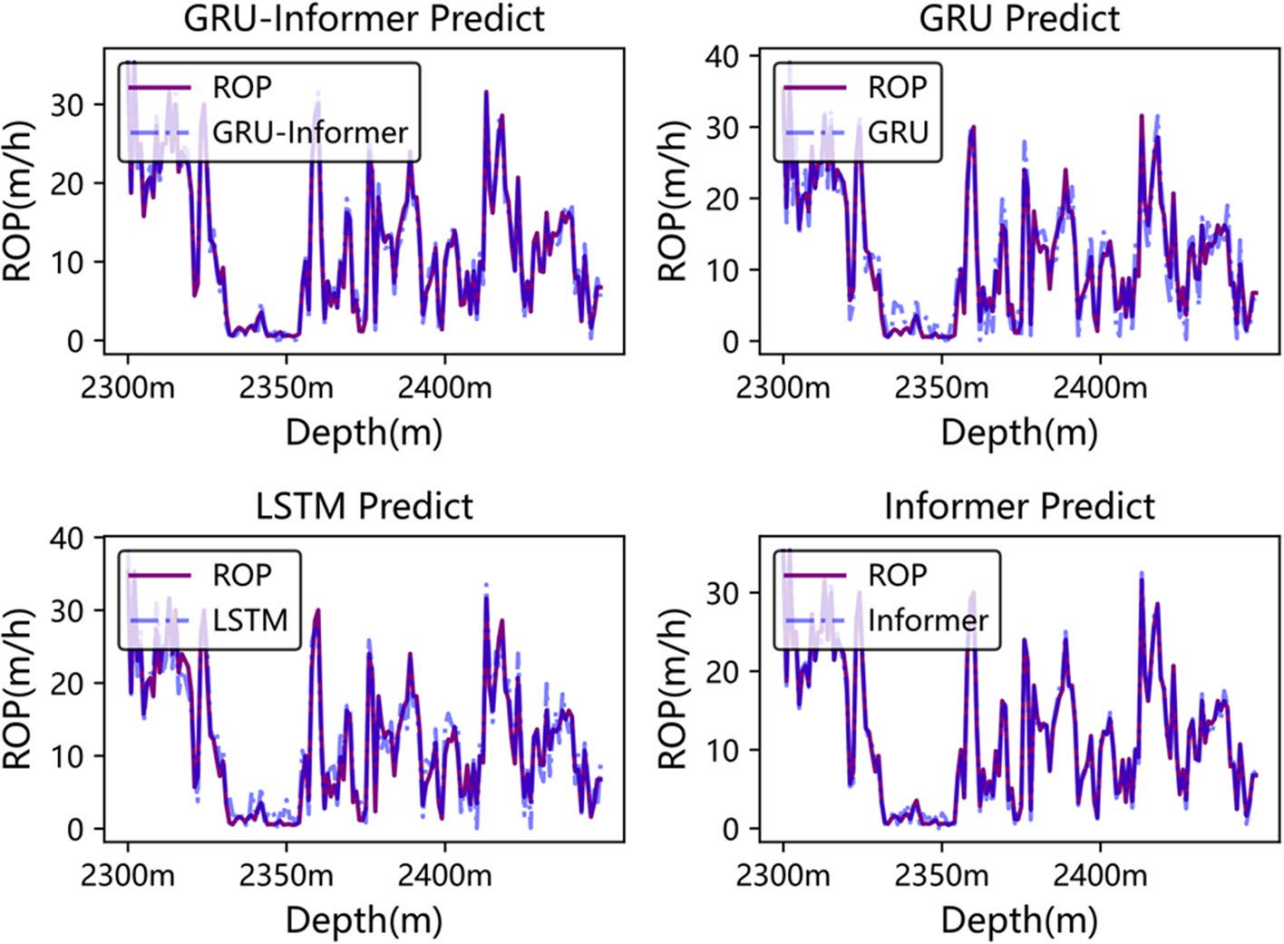
Model prediction curve of 2300–2450 m in well2.

Figures 10 and 11 depict the comparative curves of predicted and actual values of the Rate of Penetration (ROP) for each model in the well segments from 600 to 1000 m in Well3 and from 2300 to 2450 m in Well2, respectively. From Fig. 10, it can be observed that GRU-Informer performs relatively well in predicting ROP for the depth range of 600–1000 m in Well3, surpassing the GRU model, while LSTM and Informer exhibit slightly inferior performance. In Fig. 11, GRU-Informer demonstrates the most accurate prediction of ROP for the depth range of 2300–2450 m in Well2, with GRU, LSTM, and Informer also showing relatively good fitting results.

The trends of predicted values from various models in Figs. 10 and 11 are observed to be generally consistent with the actual trends of Rate of Penetration (ROP), indicating that both the GRU-Informer model and the comparative models can roughly predict the ROP trend. However, there exists a certain gap in terms of prediction accuracy. From Table 6, it is evident that the RMSE (Root Mean Square Error) values of the GRU-Informer network on the dataset are 1.16 and 0.58, respectively, lower than those of LSTM, GRU, and BP on the dataset. Furthermore, the R^2^ values of GRU-Informer on the dataset are larger than those of the other three models, being 0.96 and 0.99, respectively. This indicates that the GRU-Informer model yields predictions closely approximating the actual measured values and exhibits superior prediction accuracy compared to the other models.

**Table 6:** Evaluation index of prediction results of different models.

	Model	MAE	RMSE	R square
Well2	Informer	1.87	2.0	0.91
	LSTM	1.94	2.26	0.86
	GRU	1.97	2.28	0.85
	GRU-Informer	1.00	1.16	0.96
Well3	Informer	0.98	1.16	0.94
	LSTM	1.03	1.31	0.92
	GRU	1.10	1.81	0.90
	GRU-Informer	0.50	0.58	0.97

## Conclusion

Based on the characteristics of drilling data, this study devised the GRU-Informer model for ROP prediction. GRU utilizes gate mechanisms to manage information flow, effectively addressing the vanishing gradient issue found in traditional recurrent neural networks. Additionally, the self-attention mechanism within the Informer automatically captures complex relationships between parameters, proving particularly beneficial in the prediction of lengthy sequences. Following data cleaning and correlation analysis, input parameters were carefully selected for training. The data dimensionality was reduced from 12 to 10 dimensions, which has, to a certain extent, reduced the time required for model training, thus accelerating the model's convergence speed. Taking various factors into account, the GRU-Informer model exhibits outstanding performance in ROP prediction, and it is stable. Therefore, it holds practical significance, ultimately improving the drill bit's performance, drilling efficiency, and reducing the overall drilling project costs.

## Data Availability

Thank you very much for the attention and recognition given by the editors to our research work. We appreciate the data sharing request from the SCI journal. However, due to confidentiality agreements in our laboratory, we are unable to provide the raw data. If editors and reviewers have specific questions regarding the data, we will make every effort to provide more detailed information for explanation and clarification. For anyone interested in obtaining data from this research, please contact Dr. Bai at baikai@yangtzeu.edu.cn.
